# Statistical modeling-approach for optimization of Cu^2+^ biosorption by *Azotobacter nigricans* NEWG-1; characterization and application of immobilized cells for metal removal

**DOI:** 10.1038/s41598-020-66101-x

**Published:** 2020-06-11

**Authors:** Abeer Abdulkhalek Ghoniem, Noura El-Ahmady El-Naggar, WesamEldin I. A. Saber, Mohammed S. El-Hersh, Ayman Y. El-khateeb

**Affiliations:** 1 Microbial Activity Unit, Department of Microbiology, Soils, Water and Environment Research Institute, Agricultural Research Center, P.N., 12112 Giza, Egypt; 2 Department of Bioprocess Development, Genetic Engineering and Biotechnology Research Institute, City for Scientific Research and Technological Applications, Alexandria, 21934 Egypt; 3 Department of Agricultural Chemistry, Faculty of Agriculture, Mansoura University, Mansoura, Egypt

**Keywords:** Metals, Applied microbiology

## Abstract

Heavy metals are environmental pollutants affect the integrity and distribution of living organisms in the ecosystem and also humans across the food chain. The study targeted the removal of copper (Cu^2+^) from aqueous solutions, depending on the biosorption process. The bacterial candidate was identified using 16S rRNA sequencing and phylogenetic analysis, in addition to morphological and cultural properties as *Azotobacter nigricans* NEWG-1. The Box-Behnken design was applied to optimize copper removal by *Azotobacter nigricans* NEWG-1 and to study possible interactive effects between incubation periods, pH and initial CuSO_4_ concentration. The data obtained showed that the maximum copper removal percentage of 80.56% was reached at run no. 12, under the conditions of 200 mg/L CuSO_4_, 4 days’ incubation period, pH, 8.5. Whereas, the lowest Cu^2+^ removal (12.12%) was obtained at run no.1. Cells of *Azotobacter nigricans* NEWG-1 before and after copper biosorption were analyzed using FTIR, EDS and SEM. FTIR analysis indicates that several functional groups have participated in the biosorption of metal ions including hydroxyl, methylene, carbonyl, carboxylate groups. Moreover, the immobilized bacterial cells in sodium alginate-beads removed 82.35 ± 2.81% of copper from the aqueous solution, containing an initial concentration of 200 mg/L after 6 h. *Azotobacter nigricans* NEWG-1 proved to be an efficient biosorbent in the elimination of copper ions from environmental effluents, with advantages of feasibility, reliability and eco-friendly.

## Introduction

The world population is continuously increasing, with anticipation to reach six billion by the end of 2050. Otherwise, agricultural productivity facing water shortage, depleting soil fertility and various abiotic stresses. Furthermore, the estimates revealed annual losses in agricultural land, mainly occurred through industrialization, pollution, desertification and urban development^1^. Pollution of soil and water with heavy metals is one of the major stresses that affect agricultural production, local population health, natural resources and the balance of the ecosystem^2,3^. In addition, industrial pollution from heavy metals leads to contamination of sites in a large area, resulting in significant changes in the structure and/or biosphere of the soil as a result of transition activities^4^. Heavy metals are metallic chemical elements that have relatively high density and are toxic at low concentrations. Copper is one of these metals, which is necessary as trace element for growth and metabolic processes in living organisms, serving as an essential micronutrient ion that is involved in some metabolic processes as being a co-factor of many metalloenzymes where it plays a role in the active sites of these enzymes, but it is toxic at high concentrations^5^. Metals, including copper is present in many wastewater sources and industries including, fertilizer, wood preservatives, printing operations, paint manufacturing, copper polishing, wire drawing, electronics plating, printed circuit board manufacturing, paper and pulp, metal cleaning and refineries^6–8^. Plants are highly sensitive to the toxicity of Cu^2+^, exhibiting metabolic disorders and inhibition of growth when the amount of Cu^2+^ in the tissues increases slightly above the normal levels^9^. Otherwise, the excessive human consumption of copper in the drinking water causes gastrointestinal irritation, headaches, central nervous disorders accompanied by depression, hepatic and kidney damage, extensive capillary damage, strong mucosal irritation, stomach cramps, diarrhea, vomiting, nausea and probable liver and kidney necrotic damages^10–12^. Therefore, it is mandatory to remove copper ions to be in allowable limits without virulence levels to organisms.

Previously, the traditional procedures for removal of heavy metals from the ecosystem may include chemical and precipitation treatment, adsorption techniques, ion exchange and electrochemical and oxidation processes. These previous techniques were found to generate toxic products, too expensive and energy requirements. Generally, the bioremediation process is an eco-friendly technique that has emerged as a cost-effective alternative to traditional technologies. Additionally, the bioremediation process is a biological technique that relies on the use of biological materials like microorganisms in biosorption and/or the bioaccumulation of such heavy metal pollutants^13–15^. It could effectively minimize the risk of contaminants across the removal of toxic heavy metals from soil or groundwater^3^. The bioremediation process has many advantages e.g. low waste production, the safety of biological processes to the environment, low energy demand and self-sustainability^16,17^. Soil microorganisms are a new applicable strategy to remediate soil contaminant with heavy metals, since discovering the role of microorganisms in metal mobility and availability to the plants^18^. Microorganisms have a variety of mechanisms for handling high heavy metals concentrations and often are limited to one or a few metals^19^. In addition, the kinetics of microbial process for heavy metals removal are depending upon the complexation, metal ions reduction, efflux or as acting as electron acceptors in anaerobic respiration^16,20^. For example, previous studies showed the role of mycorrhizal fungi, N-fixing rhizobacteria and free-living rhizosphere bacteria in removing heavy metals either through direct microbial action and/or encouraging plant growth^21,22^. Further, rhizospheric bacteria increased the uptake of Cd^2+^ in *Brassica napus*^23^. The role of *Azotobacter chroococcum* with zeolite as a carrier for removing heavy metals from contaminated soil has been reported^20,24^. Another study referred to the capacity of actinobacteria in the removal of heavy metals^25^. Otherwise, El-Naggar *et al*.^26^ the efficacy of algal biomass of *Gelidium amansii* in the biosorption of the lead element from aqueous solution. Similarly, the biosorption of some heavy metals from aqueous solution has been carried out by the algal biomass^27,28^.

*Azotobacter* species are saprophytic, nitrogen-fixing bacteria that are widely distributed in soil, water and in combination with some plants. Therefore, in the present study *Azotobacter nigricans* NEWG-1 as an ecofriendly bacterium was used for the biosorption of copper ions from the medium under different growth conditions. Statistical modeling-approach was used for optimization of the biosorption process. In addition, characterization of the bacterial biomass and application of the immobilized bacterial cells for copper removal were studied.

## Results and discussion

Heavy metals represent the main pollutants in land and water resources, threatening the picture and distribution of organisms. Anthropogenic sources of metal contamination included five categories (i) metal mining, (ii) atmospheric deposition, (iii) agricultural practice (iv), industrial processes, and (v) waste disposal^29,30^. The risk factor of heavy metals can be due to accumulation in the human body across food chain^31^. The impact of heavy metals could extend to change the microbial communities and activities^32^. However, the most traditional remediation methods such as landfill, thermal treatment, acid leaching and electro-reclamation do not provide reasonable solutions due to low efficiency, costs and time consuming^33^. Remediation of heavy metals from contaminated water is a global challenge and requires new technology to enhance the process. Biological treatment was found to be more efficient and low cost^34^. One effective and promising process is phytoremediation, which is the use of plants to detoxify pollutants^28,35^. The use of the microbial approach in heavy metals leaching is another type of biological treatment, it is a promising approach since discovering the role of soil microorganisms in leaching the heavy metals^4,36^.

The present study is designed to leach copper ion by *Azotobacter nigricans* NEWG-1 using Box-Benken design as affected by the independent variables, i.e. incubation periods, pH values and Cu^2+^ concentrations.

### Cultural and morphological features of *Azotobacter* sp. strain NEWG-1

*Azotobacter* genus produces flat, slimy, paste-like colonies with a diameter of 5–10 mm. The growth is favored at a temperature of 20–30 °C. Morphological characteristics of *Azotobacter* sp. isolate NEWG-1 was observed by Gram-stained slides after incubation on nitrogen-free Asby’s medium at 30 °C for 28 h. Microscopic observation of *Azotobacter* sp. isolate NEWG-1 showed oval or spherical large bacteria (1–2 μm in diameter) that form thick-walled cysts and may produce large quantities of capsular slime. Morphological examination revealed that the *Azotobacter* sp. isolate NEWG-1 is Gram-negative, aerobic, free-living, heterotrophic bacteria, and fix atmospheric nitrogen so it can grow in the absence of nitrogen such as the modified nitrogen-free Asby’s medium.

### rRNA gene sequence analysis and phylogenetic analysis

**Figure 1:**
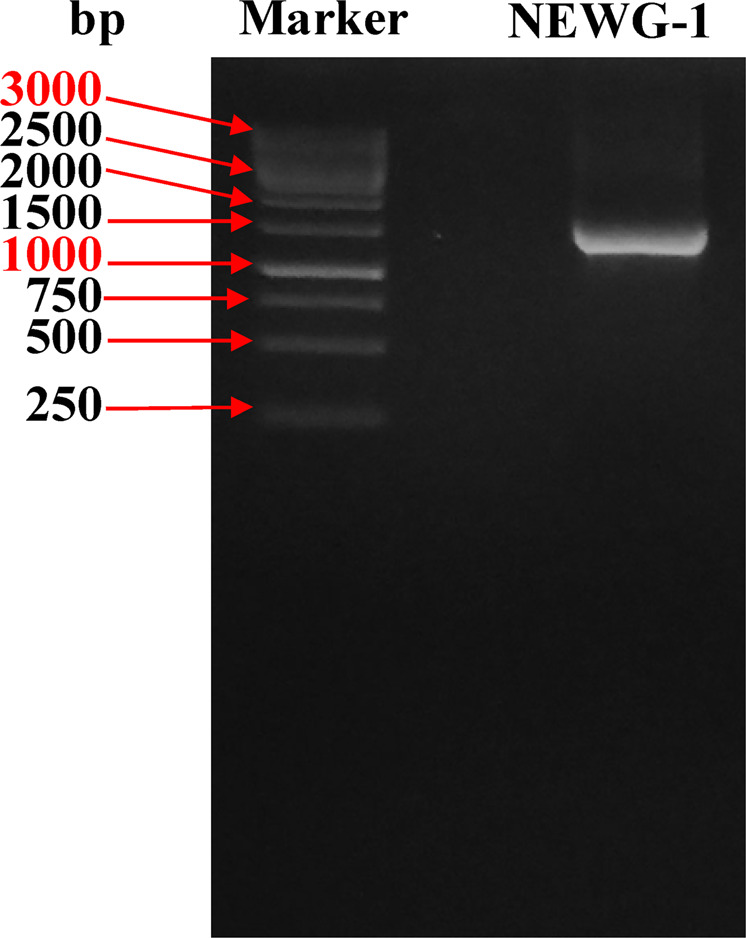
Agarose gel electrophoresis showed the PCR product of the amplified *Azotobacter nigricans* NEWG-1 16S rRNA fragment.

**Figure 2:**
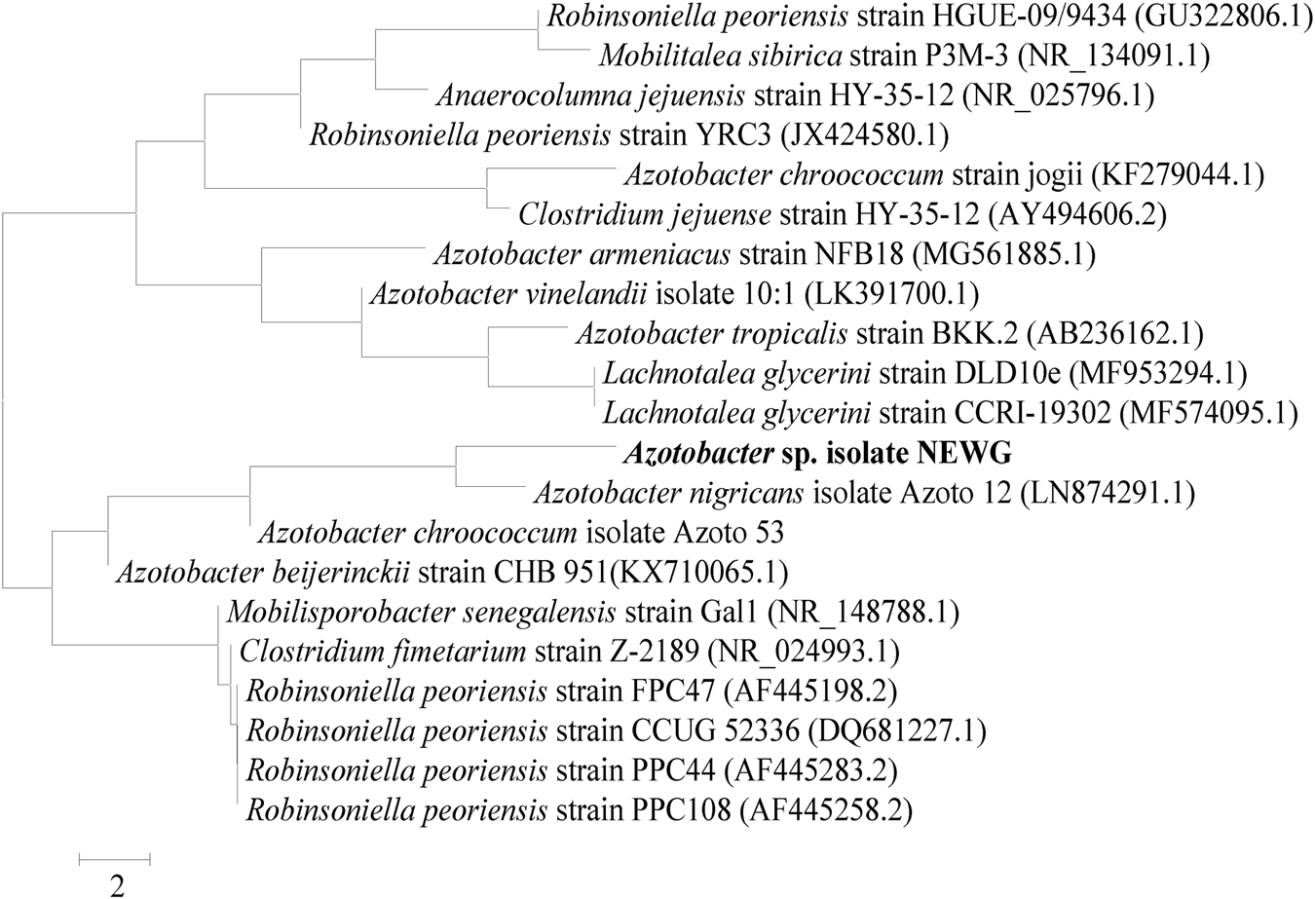
Phylogenetic tree obtained by neighbor-joining analysis showing *Azotobacter nigricans* NEWG-1 position within the genus *Azotobacter*.

The obtained 16S rRNA sequence of *Azotobacter* sp. isolate NEWG-1 was determined and the amplified 16S rRNA fragment gave sequence with 1500 bp (Fig. 1). The obtained 16S rRNA sequence was subjected to the BLAST search^37^ of the GenBank database. The phylogenetic tree (Fig. 2) was constructed using MEGA 3.0 software^38^. Accordingly, *Azotobacter* sp. isolate NEWG-1 was identified as *Azotobacter nigricans* NEWG-1 and the 16S rRNA sequence had been deposited in the DDBJ/EMBL-Bank/GenBank database under the accession number LC485953.

### Optimization of medium condition using Box-Benken

The design matrix of Box-Behnken was employed to determine the interactive effects of the independent variables, i.e. incubation periods, initial culture pH, and initial concentration of Cu^2+^ in culture medium growth to obtain the maximum copper removal% by *Azotobacter nigricans* NEWG-1. The experimental design matrix of Box-Behnken with 15 runs and the corresponding actual and predicted values of Cu^2+^ removal % were introduced in Table 1. Data obtained illustrate the variations in Cu^2+^ removal %, in which it ranged from 12.12 to 80.56%. These variations indicated that the process of optimization was important for the maximization of the removal efficiency of copper by *Azotobacter nigricans* NEWG-1. Data also show the highest value of Cu^2+^ removal (80.56%) occurred in run no.12. Whereas, the lowest value of Cu^2+^ removal was in run no.1, being 12.12%. As can be seen, the predicted values of Cu^2+^ removal are very close to the corresponding actual values, reflecting the accuracy of such model in the prediction of the response.

**Table 1:** Experimental fermentation conditions of the tested variables based on Box-Behnken design and the corresponding observed and fitted Cu^2+^ removal % by *Azotobacter nigricans* NEWG-1.

Std	Run	Tested variables						Cu^2+^ removal (%)		Residuals
		Incubation time (X_1_)		pH (X_2_)		CuSO_4_ concentration (X_3_)				
		Coded	Actual (days)	Coded	Actual	Coded	Actual (mg/L)	Experimental	Predicted	
9	1	0	4	−1	5.5	−1	50	14.73	15.16	−0.43
3	2	−1	2	1	8.5	0	125	52.58	55.69	−3.11
15	3	0	4	0	7	0	125	60.31	58.89	1.42
6	4	1	6	0	7	−1	50	50.19	52.87	−2.68
13	5	0	4	0	7	0	125	59.57	58.89	0.68
7	6	−1	2	0	7	1	200	70.88	68.20	2.68
14	7	0	4	0	7	0	125	56.8	58.89	−2.09
1	8	−1	2	−1	5.5	0	125	12.12	14.97	−2.85
4	9	1	6	1	8.5	0	125	67.47	64.62	2.85
8	10	1	6	0	7	1	200	60.32	63.60	−3.28
12	11	0	4	1	8.5	1	200	80.56	80.13	0.43
2	12	1	6	−1	5.5	0	125	18.68	15.57	3.11
5	13	−1	2	0	7	−1	50	42.02	38.74	3.28
10	14	0	4	1	8.5	−1	50	72.37	72.54	−0.17
11	15	0	4	−1	5.5	1	200	47.9	47.74	0.16

### Evaluation of Box-Behnken results

The experimental data of Box-Behnken results was subjected to the statistical analysis and ANOVA. As shown in Table 2, the overall model *F*-value=43.5. Model *F*-value is calculated as the ratio of mean square regression and mean square residual, the higher the *F*-value, the higher the significance of the model. The *P*-value of the overall model is very low (0.0003), this implies that the model is significant. The lower *P*-value (≤0.05) means the higher significance of the model. Another indication of the adequacy of the model is the non-significant lack of fit (*P*-value = 0.128), which is a prerequisite for the fit of the overall model.

**Table 2:** Analysis of variance for the response surface of Cu^2+^ removal % by *Azotobacter nigricans* NEWG-1 obtained by the Box-Behnken design.

Source of variance		Degrees of freedom	Contribution, %	Sum of square	Mean of square	*F*-value	*P*-value	Coefficient estimate
Overall model		9	98.7	6163.84	684.87	43.49	0.0003^*^	58.89
Linear effect	X_1_	1	0.7	45.41	45.41	2.88	0.1502	2.38
	X_2_	1	64.6	4029.78	4029.78	255.92	<0.0001^*^	22.44
	X_3_	1	12.9	807.02	807.02	51.25	0.0008^*^	10.04
	Total linear	3	78.2	4882.2	1627.4	103.4	0.000^*^	
Square effect	X_1_^2^	1	5.1	340.93	340.93	21.65	0.0056^*^	−9.61
	X_2_^2^	1	8.7	494.41	494.41	31.40	0.0025^*^	−11.57
	X_3_^2^	1	2.6	159.30	159.30	10.12	0.0245^*^	6.57
	Total square	3	16.4	1020.6	340.2	21.6	0.003^*^	
Interaction effect	X_1_X_2_	1	0.3	17.35	17.35	1.10	0.3420	2.08
	X_1_X_3_	1	1.4	87.70	87.70	5.57	0.0647	−4.68
	X_2_X_3_	1	2.5	156.00	156.00	9.91	0.0254^*^	−6.25
	Total interaction	3	4.2	261.1	87.0	5.5	0.048^*^	
Error effect	Pure Error	2	0.1	6.85	3.42			
	Lack-of-Fit	3	1.2	71.88	23.96	7.0	0.1276	
	Total error	5	1.3	78.7	15.8			
Overall total		14	100.0					
R^2^	98.74%	Std. Dev.	3.97					
Adj R^2^	96.47%	Mean	51.10					
Pred R^2^	81.33%	C.V.%	7.77					
Adeq Precision	20.1125	PRESS	1165.54					

*Indicates significant effect. “Std. Dev. is the standard deviation, the coefficient of determination (R^2^), Adj R^2^ is the adjusted-R^2^, Pred R^2^ is the predicted-R^2^ and PRESS is the prediction error sum of squares, Adeq Precision is adequate precision, C.V is the Coefficient of variation”.

The *P*-values were also used as a measure to verify the significance of each variable which, in fact, is necessary to understand mutual interaction patterns between the test variables. Among the test variables, the *P*-values of X_2_ (initial culture pH) and X_3_ (initial concentration of Cu^2+^) were significant, whereas, X_1_ (incubation period) was not significant. Regarding the square effect, all the variables were significant. Finally, X_2_X_3_ was the only significant interaction. The total effects of linear, square and interaction were found to be significant, being 0.000, 0.003 and 0.048; respectively.

**Figure 3:**
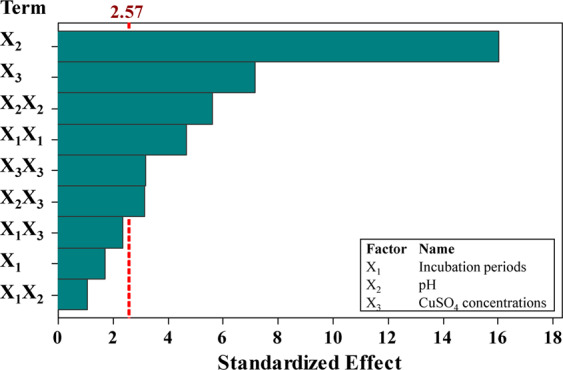
Pareto chart of the standardized effects of the tested parameters on Cu^2+^ removal % by *Azotobacter nigricans* NEWG-1.

These data were confirmed by the Pareto chart (Fig. 3), which was used to determine the contribution of each variable to the variability in the Cu^2+^ removal. The absolute values of the effects were plotted in descending order. The significance of the individual effect of each of the tested variables at the level of 0.05 was designated using the reference line on the chart. In these results, the incubation period had no significant effect on Cu^2+^ removal, on the other hand, pH followed by initial Cu^2+^ were the most efficient variables.

In order to assign the fitness of the model based on the experimental data, further statistics parameters were calculated. Data standard deviation measured a low value of 3.97. The lower the value of the standard deviation the better the model describes the response. The value of the prediction error sum of squares (PRESS) is 1165.54. A smaller value of PRESS indicates the model’s has better predictive efficiency.

Regarding the coefficient of determination (R^2^), it was found to be 98.74%. R^2^ is the variation that occurs in the response when applying the model. It is one of the important measures that usually used to determine how well the model fits the data (copper removal). The higher R^2^ value means that the model better explains the data. The adjusted-R^2^ explains the variance in the response as affected by the independent variables. However, the present adjusted-R^2^ value was 96.47%, this is high enough to explain the response as a result of the model. The predicted-R^2^ was calculated in order to estimate how well the model predicts the response for new experiments. The predicted-R^2^ of the current model was estimated to be 81.33%. Models with higher values of predicted-R^2^ have greater ability to predict the response.

**Figure 4:**
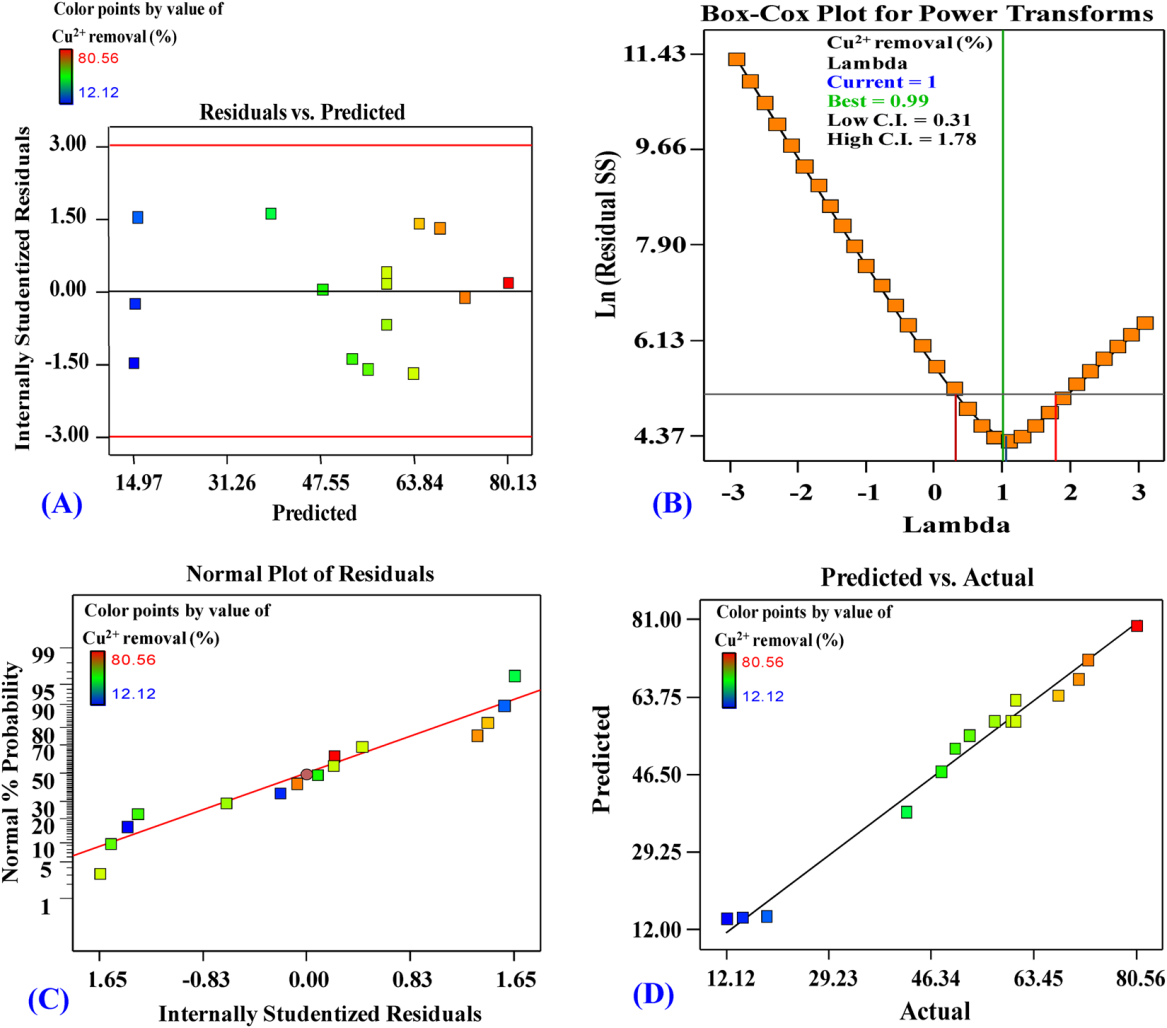
(**A**) Correlation between the residual and predicted values, (**B**) Box-Cox plot, (**C**) The normal probability plot of the residuals, (**D**) Correlation between the experimental and predicted values for Cu^2+^ removal by *Azotobacter nigricans* NEWG-1 determined by the second-order polynomial equation.

To verify that the model is adequate to meet the assumptions of the analysis, some analytical statics were performed (Fig. 4). The residuals were plotted against fitted values (Fig. 4A), the residuals on the plot fall randomly around the center line and no trends or patterns could be observed when displayed against the fitted values of Cu^2+^ biosorption. The residuals not correlated and scattered randomly and independently on both sides of the center line, indicating that residuals have constant variance and the model is adequate and meets the assumptions of the analysis.

In Box-Cox plot (Fig. 4B), the current lambda (λ) was equal to 1.0, the best λ was 0.99, whereas the confidence interval (C.I.) was between low (0.31) and high (1.78), since the best value of lambda lies between the points of low and high C.I. so, there was no recommended transform of the data. The normal probability plot (Fig. 4C) indicates that the residuals follow a straight line, meaning that the data were normally distributed. In consent with the Box-Cox plot, no needs for data transform. Plotting of the actual versus the predicted response values (Fig. 4D) showed that the data points split evenly along the 45-degree line. This can help detect the value(s) that are not easily predicted by the model.

**Figure 5:**
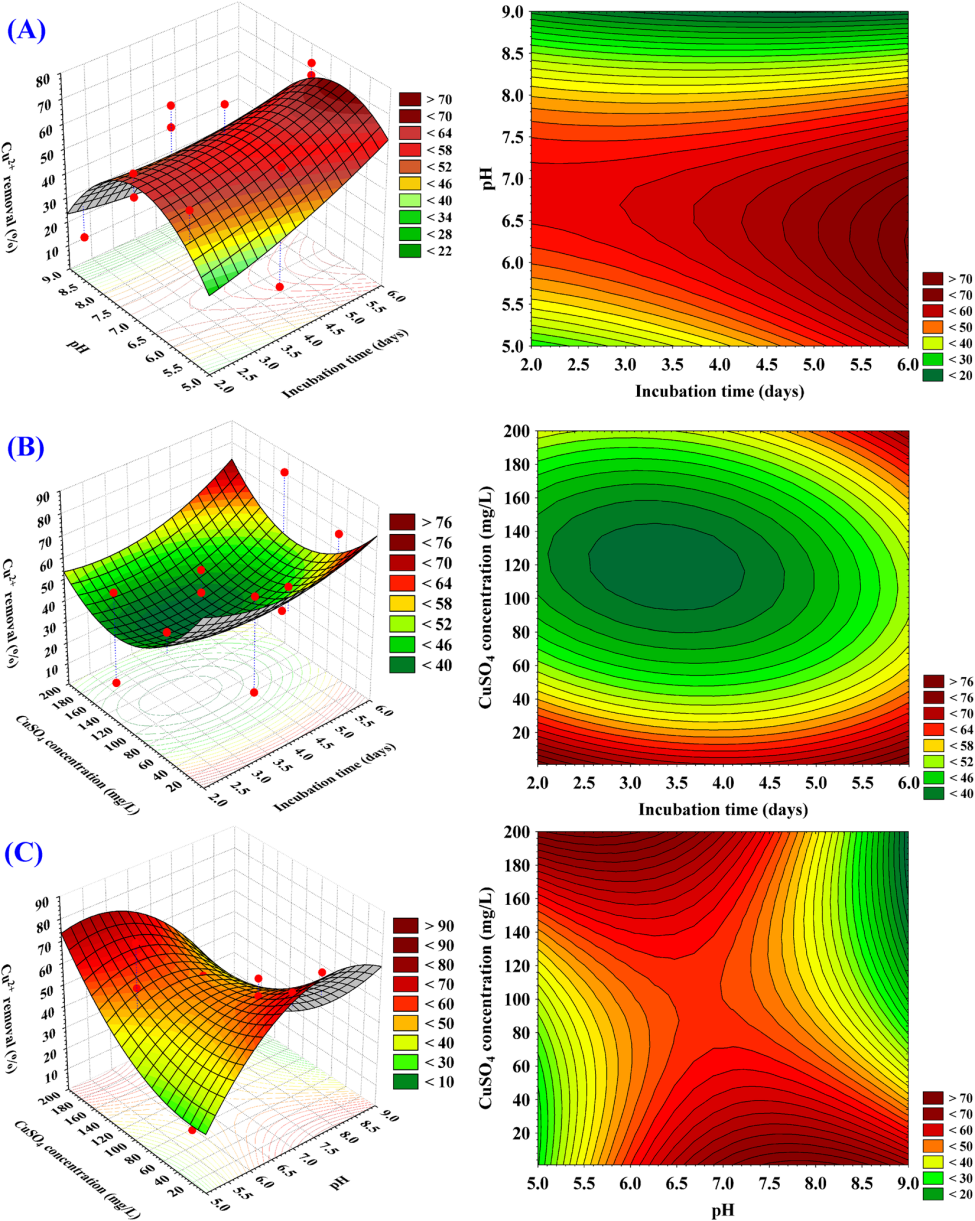
Three-dimensional surface plot of Cu^2+^ removal % by *Azotobacter nigricans* NEWG-1, showing the interactive effects of two variables at a time of the three tested variables factors, holding the third factor at the center point.

According to the previous ANOVA, the second-order polynomial equation for Cu^2+^ biosorption by *Azotobacter nigricans* NEWG-1 was calculated as coded units as follows;

1 $${\bf{C}}{\rm{o}}{\rm{p}}{\rm{p}}{\rm{e}}{\rm{r}}\,{\rm{r}}{\rm{e}}{\rm{m}}{\rm{o}}{\rm{v}}{\rm{a}}{\rm{l}}\, \% =+58.89+2.38{{\rm{X}}}_{1}+22.44{{\rm{X}}}_{2}+10.04{{\rm{X}}}_{3}-9.61{{{\rm{X}}}_{1}}^{2}-11.57{{{\rm{X}}}_{2}}^{2}+6.57{{{\rm{X}}}_{3}}^{2}+2.08{{\rm{X}}}_{1}{{\rm{X}}}_{2}-4.68{{\rm{X}}}_{1}{{\rm{X}}}_{3}-6.25{{\rm{X}}}_{2}{{\rm{X}}}_{3}$$

where X_1_ is the coded value of incubation time, X_2_ is the coded value of initial culture pH and X_3_ is the coded value of initial concentration of Cu^2+^ Three-dimensional (3D) surface and contour plots. To understand the interactive effects of the three tested variables and the optimum values of each variable required for the maximum Cu^2+^ removal %, the 3D surface and contour plots were established by plotting Cu^2+^ removal % on the Z-axis against two of the variables and the third variable is holed at the center point (shown in Fig. 5A–C). Figure 5A represents the Cu^2+^ removal % as the simultaneous effect of incubation time (X_1_), pH (X_2_) while the initial concentration of CuSO_4_ was kept at the central point (125 mg/L). The Cu^2+^ removal % increased with the increment of incubation time and with an increase in initial pH, the Cu^2+^ removal % increased beyond pH 7. A further rise in the initial pH leads to a gradual reduction in Cu^2+^ removal %. By solving the Eq. (1), the highest Cu^2+^ removal % of 70.28% could be reached using the optimal predicted levels of incubation time of 4.43 days, initial pH of 8.5 when the initial concentration of CuSO_4_ was kept at 125 mg/L.

The variations in Cu^2+^ removal (%) efficiency by *Azotobacter nigricans* NEWG-1 may be influenced by independent variables, which effect on biosorption process. Park *et al*.^39^ reported that the physicochemical factors have an influence on the biosorption performance. Moreover, Wang^40^ studying the biosorption process of copper ion by *Saccharomyces cerevisiae*. The efficacy of Cu^2+^ removal enhanced from 1 to 20 mg/L, while it was decreased in the concentrations range from 25 to 50 mg/L. The pH value was found to have a positive effect on the biosorption process. Another suggested mechanism for copper removal throughout the process of adsorption is the ion-exchange. Additionally, the cell surface and mucus layer also play critical role in both absorption and adsorption processes of metal-ions through which, the functional-groups that occurred onto cell surface make complexes with the metal-ions to act as organization atom of leaching process^41^. Besides, negative charges of the carboxyl anionic groups and phosphoric acid on the microbial cell wall surface, allows the metal ions to attach and/or pass across the cell membrane^42^. Moreover, the pH values influence the sites of sorbate^43^ through the competition that occurs among metal ions and protons.

Rohini and Jayalakshmi^44^ reported a bioremediation process of copper ions using *Bacillus cereus* with a maximum tolerable capacity of 600 mg/L copper under optimal growth conditions. Whereas, Pugazhendhi *et al*.^45^ reported the efficacy of *Ralstonia solanacearum* for biosorption of lead from aqueous solution.

The 3D surface and contour plots in Fig. 5B illustrate Cu^2+^ removal % as a function of incubation time (X_1_) and initial concentration of CuSO_4_ (X_3_) while pH (X_2_) was fixed at 7. Figure 5B indicates that low Cu^2+^ removal % is supported by lower incubation time (X_1_), the increase in incubation time, the Cu^2+^ removal % increases and the maximum Cu^2+^ removal % obviously obtained at near 4 days of incubation. On the other side, the initial concentration of CuSO_4_ (X_3_) up to 200 mg/L had a steady positive effect on the Cu^2+^ biosorption by *Azotobacter nigricans* NEWG-1. By analysis of Fig. 5B and solving the Eq. (1), the maximum predicted Cu^2+^ removal % of 75.64% could be reached using pH 7, 200 mg/L of the initial concentration of CuSO_4_ and incubation period of 3.8 days.

However, the increases of metal ions could affect the quantity of the bio-absorbed heavy metal per unit weight of biomass^26^. Another study suggesting that the leaching process falls into two categories (i) biosorption (passive) using non-living cells (ii) bioaccumulation using living cells^46,47^. However, another investigation pointed out that the leaching process depends upon the covalent interaction of metal ion on the cell surface or inside the cell by different methods^48^. The adsorption ability of species of *Azotobacter*, *Pseudomonas*, *Serratia* and *Klebsiella* to heavy metals has been reported^49^. Further, the copper ions were found to be utilized in the metabolic process of bacteria, e.g. cytochrome c oxidase^50^. Moreover, leaching of Cr(VI) using fungal biomass has been investigated^51^.

Figure 5C shows Cu^2+^ removal % as influenced by pH (X_2_) and initial concentration of CuSO_4_ (X_3_) by maintaining the incubation time (X_1_) at 4 days. With an increased concentration of CuSO_4_; Cu^2+^ removal % by *Azotobacter nigricans* NEWG-1 was increased and the maximum Cu^2+^ removal % has been obtained at a high level of CuSO_4_. The Cu^2+^ removal % increases with the increment of pH and the maximum Cu^2+^ removal % obviously obtained at pH 8 and the further increase in pH led to a decrease in Cu^2+^ removal %. By analysis of Fig. 5C and solving the Eq. (1), the maximal predicted Cu^2+^ removal % of 81.19% could be reached using 4 days of incubation time and the optimal predicted levels of pH 8 and initial concentration of CuSO_4_ of 200 mg/L.

### Model verification

In order to determine the optimal combination of the tested variables, that maximize the Cu^2+^ biosorption, the response optimization was carried out to increase the efficacy of *Azotobacter nigricans* NEWG-1 in Cu^2+^ biosorption. The optimal predicted levels of 4 days of the incubation time, pH 8 and 200 mg/L of initial CuSO_4_ to maximize the efficacy of Cu^2+^ biosorption in the fermentation medium were estimated. The theoretical estimated Cu^2+^ biosorption based on the equation of the quadratic model at such condition was 81.19%. In order to confirm the optimization results, the theoretical calculations from the regression equation were laboratory validated using the estimated levels of the tested variables, the laboratory value of Cu^2+^ biosorption was found to be 80.56%. This value is closely related to the theoretical value, confirming the accuracy of the proposed model.

In this study, the maximum Cu^2+^ removal by *Azotobacter nigricans* strain NEWG-1 was 80.56% under the conditions of 200 mg/L CuSO_4_, 4 days incubation period and pH 8.

Narasimhulu^52^ used response surface methodology to optimize the process variables for batch biosorption of Cu^2+^ by *Bacillus subtilis*. The optimum levels of process variables for the highest biosorption of Cu^2+^ (78.4%) by *Bacillus subtilis* were determined to be contact time of 30 min, biomass concentration of 2 mg/mL, pH of 4 and temperature of 32 °C using CuSO_4_ at an initial concentration of 10 mg/L. On the other hand, Rajeshkumar and Kartic^53^ used one factor at a time optimization method to determine the optimal values of various physicochemical variables on the biosorption of Cu^2+^ by *Bacillus* sp., the physicochemical variables for maximum biosorption of Cu^2+^ (88%) were pH (8), temperature 35°C when the initial Cu^2+^ concentration was 100 mg/L. Moreover, Choińska-Pulit *et al.*^54^ used Box-Behnken design to optimize the biosorption process of Cu^2+^ by *Pseudomonas azotoformans* JAW1 in an aqueous medium. The maximum biosorption of Cu^2+^ by *Pseudomonas azotoformans* JAW1 (63.32%) was achieved at the optimum levels of process variables (concentration of the biosorbent of 2 g/L, the initial metal concentration of 25 mg/L and pH 6). Also, El-Ahwany^55^ used Plackett-Burman experimental design in 12 experimental runs to evaluate the significance of 11 process variables on Cu^2+^ biosorption by *Oenococcus oeni* PSU1. The most significant variables were mixing speed, immobilization and initial copper concentration. These variables were selected and further optimized using the three-level Box–Behnken design to determine the optimal level each. The estimated optimal levels of these three variables for maximum Cu^2+^ biosorption by *Oenococcus oeni* PSU1 (85%) were immobilization (2.27%), mixing speed (136 rpm) and metal concentration (8.6 mg/mL).

Manohari and Yogalakshmi^56^ studied the Cu^2+^ ions removal process by genera of *Bacillus* and *Arthrobacter*, using Box-Behnken design. Three variables (pH, temperature and Cu^2+^ concentration) were selected to find out the optimal levels and to evaluate the combined effects of these variables on Cu^2+^ removal efficiency by isolated bacterial consortium^2^. The optimal values of the selected variables for the highest Cu^2+^ bioremoval of 82.8% were 168 h of incubation at a temperature of 32.5°C, pH 5 and 600 mg/L copper concentration^2^. On the other hand, Ghosh and Saha^57^ used central composite design to optimize the bioremediation process variables and to study the effects and interactions of these variables on Cu^2+^ removal from aqueous solution by *Stenotrophomonas maltophilia* PD2. Initial pH, initial copper concentration and contact time on removal % of Cu^2+^ was studied using central composite design. The maximum experimental Cu^2+^ removal % by copper-resistant *Stenotrophomonas maltophilia* PD2 (90%) was obtained at the optimal levels of initial copper concentration (50 mg/L), contact time (26 h) and pH (5.5). The efficiency of leaching was 63.32%. As well as, the bacterial organism, *Bacillus licheniformis* has the ability to remove 80% copper ion under pH 6.5 and contact time of 216 h^58^. In the context, the studies of Karthik *et al*.^59,60^ reported the removal of Cr(VI) by using *Cellulosimicrobium funkei* strain AR8.

### Fourier transform infrared (FTIR) analysis

**Figure 6:**
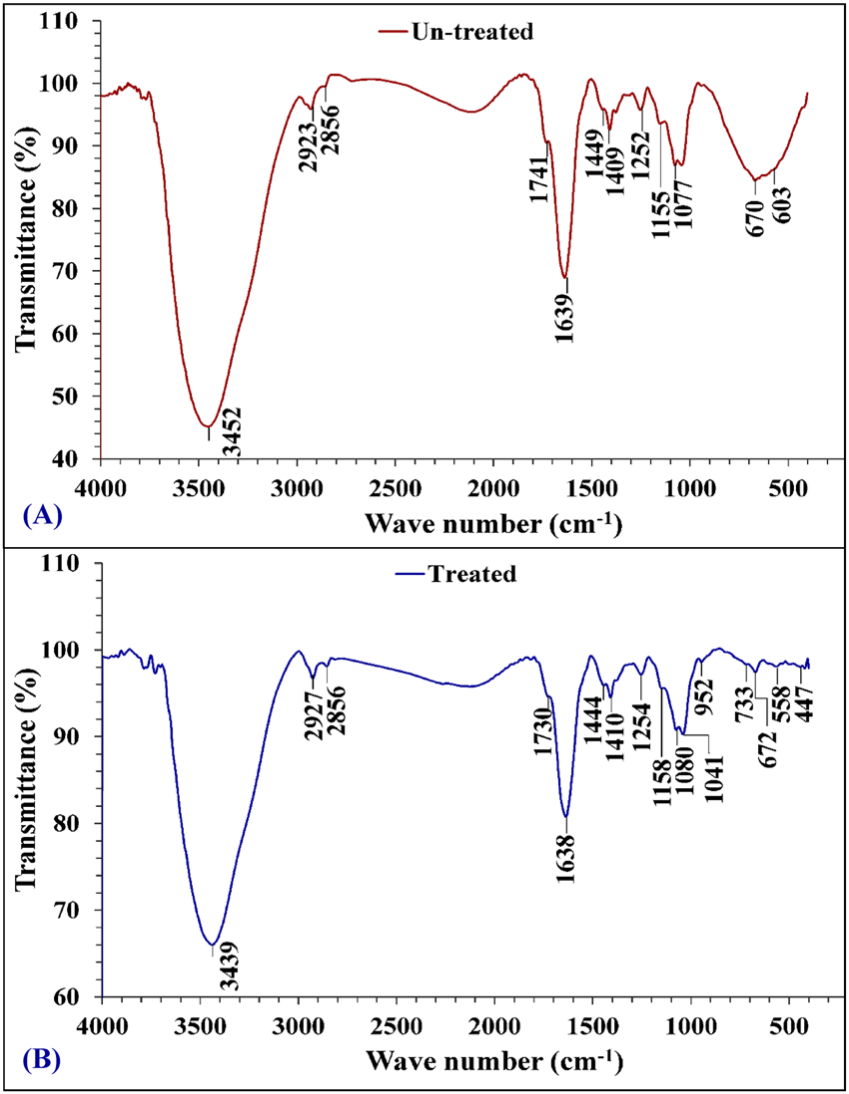
FTIR analysis of *Azotobacter nigricans* NEWG-1 cells: (**A**) before and (**B**) after biosorption of Cu^2+^ ions from aqueous solution.

Dried cells of *Azotobacter nigricans* NEWG-1 were analyzed before and after copper biosorption (Fig. 6) to investigate the interaction of copper ion with functional groups of the cells wall of the bacterium. Several functional groups have the ability to bind with metal ions, such as amino, phosphate, carboxylate and hydroxyl groups^61^. Biosorption of metal ions takes place via the ion exchange process on the cell surface. The adsorbent spectra were measured in the range between 400 and 4000 cm^−1^ wave number^62^. The chart of analysis indicates that the spectra of 3452 and 3439 cm^−1^ O-H stretching vibrations. 2923 and 2927 cm^−1^ (asymmetric CH_2_ stretch), it is probable that the band observed at 2856 cm^−1^ is the difference between the hydroxyl stretching vibrational frequency and the hydroxyl translation frequency. 1741, 1730 cm^−1^ is the wavenumber for C=O in the carboxyl group (-COOH) or carbonyl ester 1638, 1639 cm^−1^ is most likely due to the presence of a C=O stretch in an amide bond. A band appeared at 1634 cm^−1^ was assigned to C=N stretching vibrational band.1449, 1444 cm^−1^ (CH_2_ bending vibration). 1409 cm^−1^ (COO-) carboxylate group. 1410 cm^−1^ (C-O). The bands at 1155 and 1158 cm^−1^ are due to the C-O stretching, whereas bands at 1077 and 1041 cm^−1^ are due to strong C-O stretching (primary alcohol). 670 cm^−1^ strong cis-disubstituted alkenes. Bands at 670, 672 cm^−1^ and around 447 cm^−1^ indicate the bidentate ligand, while the absence of the band at 670-630 cm^−1^ reveals monodenticity. 558 cm^−1^ was assigned to CuO stretching vibrations. Copper –oxygen (Cu-O) stretching bands have been notable at 447 cm^−1^. The lower frequency regions of IR spectra of all complexes recorded weak bands around 447-558 cm^−1^ is attributed to Cu-N bonds^63^.

### Scanning electron microscopy (SEM)

**Figure 7:**
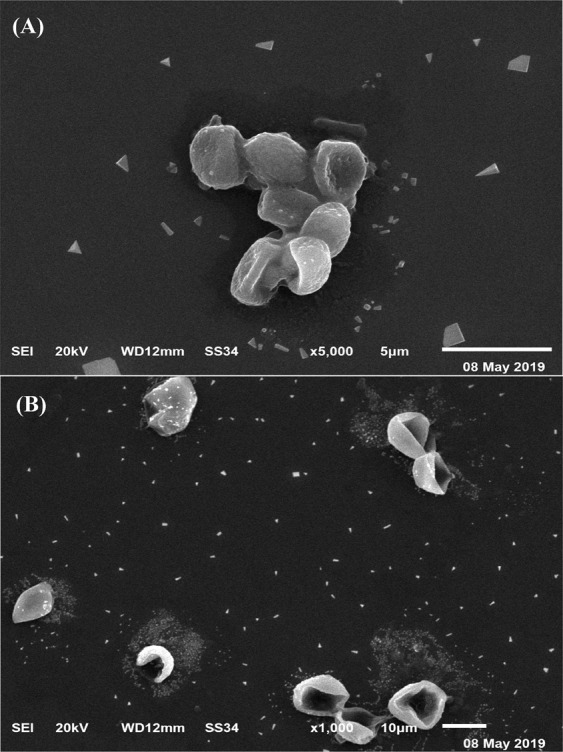
SEM micrograph of *Azotobacter nigricans* NEWG-1 cells: (**A**) before and (**B**) after biosorption of Cu^2+^ ions from aqueous solution.

Figure 7 shows a micrograph of *Azotobacter nigricans* NEWG-1 cells before and after the adsorption of copper ions. It is obvious that the normal, non-treated cells have a regular shape. They are oval or spherical large cells with a thick-wall and large slime capsular (Fig. 7A). On the other side, after the bacterial adsorption of copper ions, Fig. 7B showed dispersed cells with irregular shapes, probably due to the toxicity of the copper ions on the cell wall. In contrast to vegetative cells, cyst formation could be also observed, which enables bacterial cells to resist adverse environmental factors such as the concentration of nutrients.

### Electron dispersive spectroscopy (EDS)

**Figure 8:**
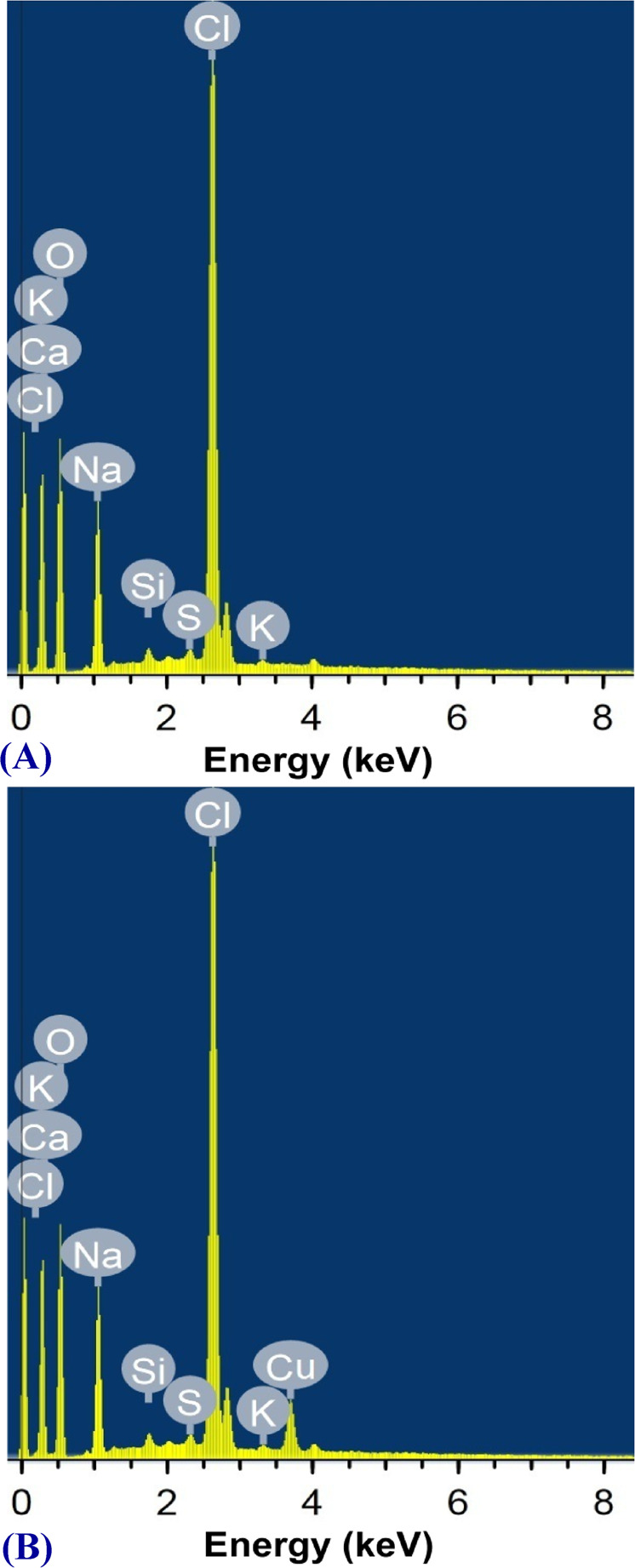
EDS analysis of *Azotobacter nigricans* NEWG-1 cells: (**A**) before and (**B**) after biosorption of Cu^2+^ ions from aqueous solution.

The EDS study was conducted in order to obtain the elementary data (Fig. 8). The EDS spectrum reveals that the binding of Cu^2+^ onto the cells surface of the tested bacterium is evident in Fig. 8B.

### Copper removal by the alginate-immobilized cells

**Figure 9:**
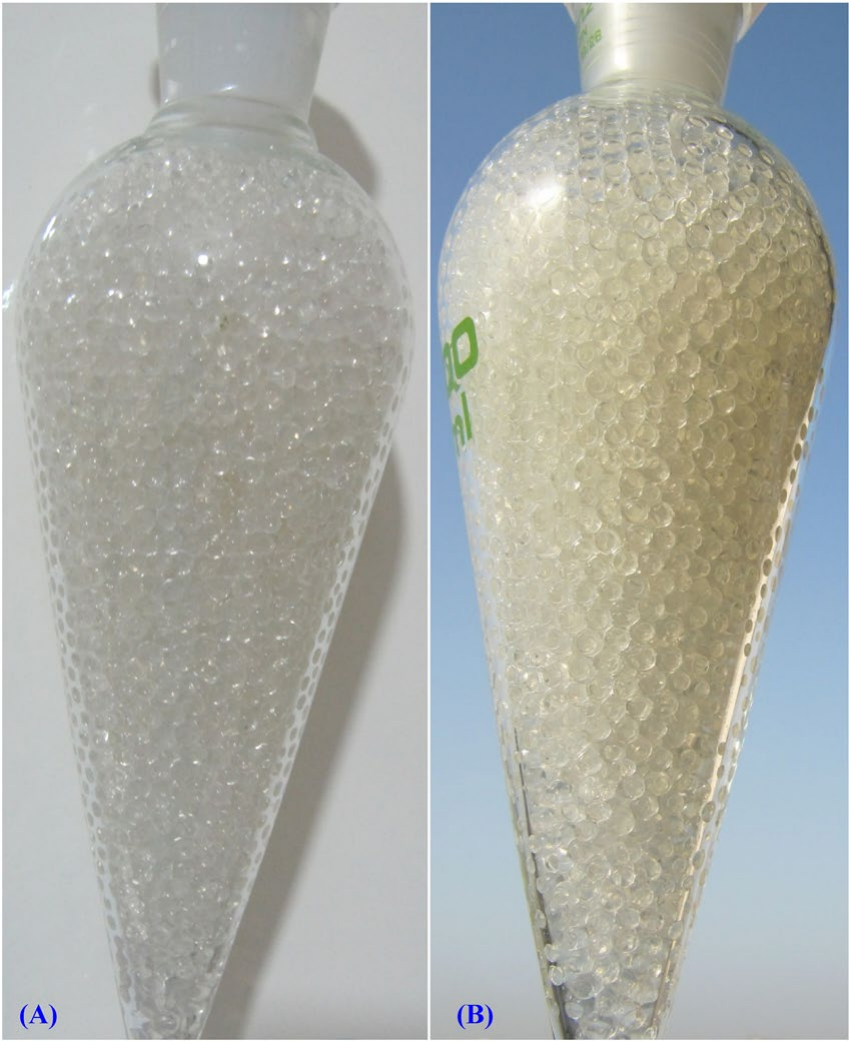
Immobilization of *Azotobacter nigricans* NEWG-1in alginate beads during Cu^2+^ ions removal from aqueous solution. (**A**) Separating funnel packed with alginate beads, (**B**) Separating funnel packed with alginate- *Azotobacter nigricans* NEWG-1 beads.

**Figure 10:**
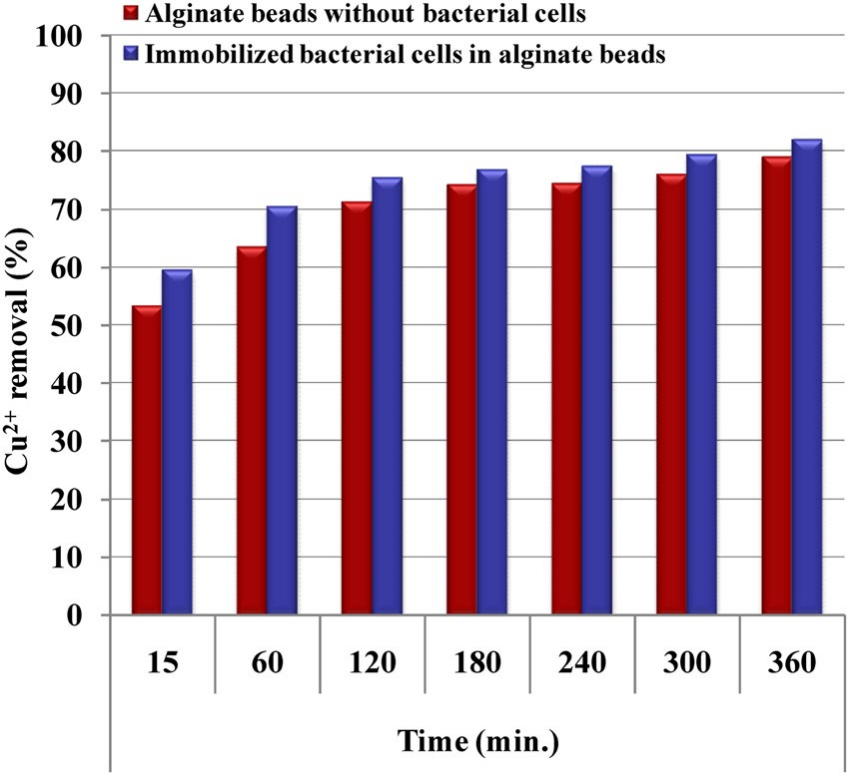
Application of immobilized *Azotobacter nigricans* NEWG-1 cells in Cu^2+^ ions removal from aqueous solution.

The ability of the immobilized *Azotobacter nigricans* NEWG-1 to remove copper ions from aqueous solution was studied, and the findings are shown in Figs. 9 and 10. The results show that the treatment of aqueous copper solution (200 mg/L) with immobilized *Azotobacter nigricans* NEWG-1 cells for 6 h resulted in the Cu^2+^ removal percentage of 82.35 ± 2.81%, which is significantly higher than those of using sodium alginate beads as a control (78%) (Fig. 10). Immobilization of *Azotobacter nigricans* NEWG-1 cells in polysaccharide beads, made of sodium alginate, could be more freely suspended cells to remove copper ions from wastewater as an alternative to current physical and chemical treatment techniques. *Azotobacter nigricans* strain NEWG-1, is an efficient and safe Cu^2+^ biosorbent, which makes possible to use the bacterium in the treatment of wastewater. The immobilized cells are more efficient in removing metals than the free cells^15,64^ and the size of the beads that are used in biomass immobilization is also a significant factor^65^.

## Materials and Methods

### Microorganism and culture maintenance

*Azotobacter* sp. was kindly obtained from the Department of Microbiology, Soils, Water and Environment Research Institute, Agricultural Research Center (Affiliation ID: 60019332), Giza, Egypt. Nitrogen-free Ashby medium consisting of (g/L) 5 glucose, 5 mannitol, 0.1 CaCl_2_·2H_2_O, 0.1 MgSO_4_·7H_2_O, 0.005 Na_2_MoO_4_·2H_2_O, 0.9 K_2_HPO_4_, 0.1 KH_2_PO_4_, 0.01 FeSO_4_·7H_2_O, 5 CaCO_3_, 15 g agar, pH 7.3 was used for the maintenance for the bacterial strain^66^. The bacterium was grown on slants of the medium supported with 15 g for 72 h, and sub-cultured periodically^66^. The sterilization was carried out at 121 °C for 20 min.

### Identification of *Azotobacter* sp. NEWG-1

#### rRNA sequencing

DNA extraction for the bacterial sample was performed using the thermo Gene JET Genomic DNA Purification Kit (#K0721). The PCR reaction and sequencing were performed according to the method of El-Naggar *et al*.^67^.

#### Inoculum preparation

For inoculum preparation, the bacterium was grown on the Asby’s medium under shaking at 30 °C for 72 h, the cell bacterial count was adjusted to obtain 10^8^ CFU/mL, 5% (v/w) inoculum was used to inject 50 mL of fermentation medium in 250 mL Erlenmeyer flasks.

#### Fermentation medium

Nitrogen-free Ashby medium, consisting of (g/L) 5 glucose, 5 mannitol, 0.1 CaCl_2_·2H_2_O, 0.1 MgSO_4_·7H_2_O, 0.005 Na_2_MoO_4_·2H_2_O, 0.9 K_2_HPO_4_, 0.1 KH_2_PO_4_, 0.01 FeSO_4_·7H_2_O, 5 CaCO_3_, 15 g agar, pH 7.3 was used as fermentation medium for the bacterial strain^66^.

#### Optimization of Cu^2+^ biosorption

The experiment was performed to find out a suitable mathematical model to be used to optimize the Cu^2+^ removal by *Azotobacter nigricans* NEWG-1 under liquid state fermentation conditions. The effect and interaction of the three factors; incubation time (X_1_), initial culture pH (X_2_) and initial concentration of Cu^2+^ (X_3_) in the medium were investigated using Box-Behnken experimental design. To address the best combination of the three factors, each was investigated at three levels during the fermentation, i.e. X_1_ at 2, 4 and 6 days, X_2_ at 5.5, 7.0 and 8.5 and X_3_ at 50, 125 and 200 mg/L. After performing the Box-Behnken experimental design, the residual Cu^2+^ in the fermentation medium was determined and the removal percent of Cu^2+^ was calculated and fitted to the second-order polynomial quadratic model equation:

2 $$Y={\beta }_{0}+\sum _{i}{\beta }_{i}{X}_{i}+\sum _{ii}{\beta }_{ii}{X}_{i}^{2}+\sum _{ij}{\beta }_{ij}{X}_{i}{X}_{j}$$

where; Y is the Cu^2+^ removal %, X_i_, and X_j_ are independent variables; β_0_ model constant, β_i_, is linear Coefficients; β_ii_ is the quadratic coefficients and β_ij_, is cross-product coefficients. The model was laboratory validated to ensure the fitness of the theoretically calculated value of each factor, using the previous equation.

#### Determination of Cu^2+^ bioremoval %

The solutions of Cu^2+^ have been made to reach the required concentrations in mg/L by dissolving copper (II) sulfate in 100 mL distilled water. At the end of the trials the residual copper was analyzed according to AOAC^68^ by Atomic Absorption Spectrophotometer (AAS) (Buck scientific 210 VGP, Inc.). The characteristic wavelengths were element-specific and accurate to 0.01- 0.1 nm. The apparatus has digital absorbance capable of operating at a wavelength of 324.8 nm for copper and has a detection limit of 0.005 mg/L. The line sources used in AAS is the single hollow cathode lamp. Analysis of copper was conducted by air acetylene flow FAAS. The sample absorbance displayed on a digital terminal screen. AAS was used for quantitative determination by using air acetylene flow rate of 1.0 L/min flame atomic absorption spectrophotometer for copper.

#### Experimental design and statistical analysis

The design of the Box-Behnken matrix and statistical analysis of variance (ANOVA) were performed using the statistical software packages Minitab (version 18, Minitab Inc., U.S.A.) and “Design-Expert software version 7 for Windows. The STATISTICA software (Version 8.0, StatSoft Inc., Tulsa, USA) was used to plot the 3D surface plots”.

#### FTIR spectroscopy

Before and after Cu^2+^ removal, the cells of *Azotobacter nigricans* NEWG-1 were analyzed using Fourier transform infrared spectroscopy to detect the functional groups present in the cells. With KBr pellets, the bacterial cells were implemented. The FTIR spectra of *Azotobacter nigricans* NEWG-1 were measured in the range between 400 and 4000 cm^−1^ using Thermo Fisher Nicolete IS10, “USA spectrophotometer at Spectral Analyses Unit, Faculty of Science, Mansoura University, Mansoura, Egypt”.

#### SEM investigation

To evaluate removal of the Cu^2+^ and to examine the surface of bacterial cells, dry cells of *Azotobacter nigricans* NEWG-1 (before and after removal of the Cu^2+^) were gold-coated and examined at various magnifications using SEM “scanning electron microscope, JEOL TEM-2100 attached to a CCD camera at an accelerating voltage of 200 kV at Central Laboratory, Electron Microscope Unit, Faculty of Agriculture, Mansoura University, Mansoura, Egypt”.

#### EDS evaluation

Energy-dispersive X-ray analysis was performed by means of JEOL TEM-2100 connected to a CCD camera at an accelerating voltage of 200 kV at Central Laboratory, Electron Microscope Unit, Faculty of Agriculture, Mansoura University, Mansoura, Egypt.

#### Cells immobilization

4 g sodium alginate dissolved into 100 mL of distilled water with continuous stirring for 30 min at 60 °C to prepare the solution of 4 percent sodium alginate^69^. Following cooling, the sterile sodium alginate gel was supplied with *Azotobacter nigricans* NEWG-1 cells of 10^5^ CFU/mL from 72 h grown vegetative cells with stirring for 5 min at room temperature. The beads with a diameter of 1.5 ± 0.2 mm were produced by adding drop-wise of the alginate-bacterial biomass combination into a cold sterile solution of 2.5% CaCl_2_ through 3 mL syringe with gentle stirring at room temperature to make spheres. The resulting beads were washed several times with sterilized distilled water in order to remove any residues of CaCl_2_ from the surface of the beads and then stored at 4 °C overnight in distilled water for stabilization and hardness of the beads. Similarly, sodium alginate beads were prepared without incorporating bacterial biomass and applied as control. Separating funnel experiment was conducted to determine the efficiency of the alginate-bacterial beads to remove the metal ions. The Simax glass separating funnel was packed with the alginate-bacterial beads, a metal solution has been added (200 mg/L). Samples (5 mL) were collected regularly (every 30 min) from the separating funnel effluent at a flow rate of 3 mL/min. the collected fractions were analyzed with the Atomic Absorption Spectrophotometer (AAS) (Buck scientific 210 VGP, Inc.). The difference of the metal solution concentration prior and after adsorption determined the biosorption capacity of metal ions by the bacterial cells.

## Conclusion

*Azotobacter nigricans* NEWG-1 showed high efficacy in copper removal (80.56%) after 6 h, encouraging the application of such bacterium according the proposed procedure to remove copper ions from similar natural aqueous polluted-solutions. Ultimately, offering an effective and eco-friendly procedure for the eradication of copper ions from wastewater.

## Data Availability

The Sanger sequencing data of 16S rRNA sequence had been deposited in the DDBJ/EMBL-Bank/GenBank database under the accession number LC485953.
