# Author Correction: Statistical modeling-approach for optimization of Cu^2+^ biosorption by *Azotobacter nigricans* NEWG-1; characterization and application of immobilized cells for metal removal

**DOI:** 10.1038/s41598-020-73837-z

**Published:** 2020-10-01

**Authors:** Abeer Abdulkhalek Ghoniem, Noura El-Ahmady El-Naggar, WesamEldin I. A. Saber, Mohammed S. El-Hersh, Ayman Y. El-khateeb

**Affiliations:** 1 Microbial Activity Unit, Department of Microbiology, Soils, Water and Environment Research Institute, Agricultural Research Center, P.N, Giza, 12112 Egypt; 2 Department of Bioprocess Development, Genetic Engineering and Biotechnology Research Institute, City of Scientific Research and Technological Applications (SRTA-City), Alexandria, 21934 Egypt; 3 Department of Agricultural Chemistry, Faculty of Agriculture, Mansoura University, Mansoura, Egypt

Correction to: *Scientific Reports* 10.1038/s41598-020-66101-x, published online 11 June 2020

The original version of this Article contained an error in Affiliation 2, which was incorrectly given as ‘Department of Bioprocess Development, Genetic Engineering and Biotechnology Research Institute, City for Scientific Research and Technological Applications, Alexandria, 21934, Egypt’.

The correct affiliation is listed below:

‘Department of Bioprocess Development, Genetic Engineering and Biotechnology Research Institute, City of Scientific Research and Technological Applications (SRTA-City), Alexandria, 21934, Egypt.’

This error has now been corrected in the HTML and PDF versions of the Article.

